# Survivors and the Response to the Ebola Virus Disease in the Provinces of North Kivu and Ituri in the Democratic Republic of Congo

**DOI:** 10.29245/2578-3009/2023/S3.1105

**Published:** 2023-05-12

**Authors:** Joseph Okeibunor, Tieman Diarra, Nkechi Onyeneho, Amadou Baïlo DIALLO, Michel N’da Konan Yao, Mamoudou Harouna Djingarey, Zabulon Yoti, Ibrahima Socé FALL, Dick Chamla, Abdou Salam Gueye

**Affiliations:** 1 World Health Organization, Switzerland; 2 Independent Consultant, Mali; 3 University of Nigeria, Nsukka; 4 Independent Public Health Expert, Niger

**Keywords:** Recovered, Ebola treatment center, Rumors, Stigma, Care, Integration, Support Return to family, Community welcome

## Abstract

We explored issues around the integration of survivors in communities and the implications for the Ebola Virus Disease (EVD) response in the Democratic Republic of Congo (DRC). We conducted a survey with 800 randomly selected respondents using a structured questionnaire. Respondents were persons aged 18 years and above. Focus group discussions (FGDs) and in-depth interviews (IDIs) were employed to obtain contextual data on the issues. Community leaders, health workers, and response pillar leads engaged in IDIs, while community members were involved in FGDs. The results revealed that the survivors suffered stigmatization and, upon return to the communities, were avoided by the community members due to fear of contamination. Some thought that the survivors should be supported in adjusting to the community, while some recommended engaging the survivors in EVD response activities.

## Introduction

Being cured is an expected result of treatment for the Ebola virus disease (EVD). Those who have recovered are evidence of the effectiveness of treatment, and of the importance of the work carried out by Ebola treatment centers (ETCs). Owing to the intense media coverage of people’s discharge from ETCs, the interviews were very short and limited to an assessment of their experiences in terms of treatment, food, and relations with the nursing staff. People would generally leave the ETCs being satisfied with the treatment they had received. Only a few did not appreciate the food as it differed from what they were used to.

After returning to their families, the recovered spoke more profoundly about their experience and talked about their quest for treatment of a disease they were unfamiliar with. Some of them went to hospitals, health centers, and health posts until they received a referral to an ETC. During their search for treatment, most of them had tried self-medication, traditional medicine, and modern medicine.

Once they returned to their homes, the recovered claimed to be satisfied with the treatment, attention, and care they received. They were equally satisfied with the information they received about the disease at the ETCs. This contrasted with the rumors about EVD that had caused them to dread going to an ETC in the first place. After being admitted for treatment, these people were able to dispel the rumors and inform others about the reality of ETCs. The recovered helped to spread information about the disease and its treatment.

However, the reintegration of former patients into their original environment was not always easy. Although this was not the case for all of them^1,2^, some suffered stigmatization by members of their community. This also impacted their economic activities as some patients lost clients or jobs. On an emotional level, some were abandoned by friends or lost confidence in themselves. Stigma was also a source of shame, frustration, and fear^3,4^. Nevertheless, those who recovered were satisfied with the support they received, including psychosocial assistance. This support also refers to medicine, food, and other material goods. Some of the former patients we met had responsibilities in the response teams and had created an association to better contribute toward the fight against EVD.

This article primarily deals with the discharge of those who recovered in ETCs, and their return to their communities. It then focuses on the community members’ perceptions of the patients who recovered and the stigmatization they faced. Finally, it discusses the proposals made by the recovered in terms of care. This article was built on discussions with the survivors that took place as soon as they were discharged from ETCs and returned to their families, as well as the experiences of patients documented in Guinea and Sierra Leone^1–8^.

## Study Design and Methods

### Study Design

This study was designed to explore and document experiences and lessons around the response to the 10^th^ EVD outbreak in North Kivu and Ituri provinces of the Democratic Republic of Congo (DRC). It adopted a cross-sectional design with mixed methods techniques of data collection. The cross-sectional design allows multiple windows of data harvesting, while the mixed methods technique provides benefits of both quantitative and qualitative approaches; together, they guarantee the integrity, robust interpretation, and conclusion that this type of evaluation deserved.

#### Selection of Study Area and Population

The study was carried out in North Kivu and Ituri provinces where the 10^th^ EVD outbreak occurred in the DRC.

**Ituri** is one of the 26 provinces of the DRC. Its capital is the city of Bunia. The Ituri Rainforest is in this area. It is located northeast of the Ituri River and on the western side of Lake Albert. Ituri is a region of high plateau (2000–5000 meters) that has a large tropical rainforest and the landscape of savannah. The district has rare fauna, including okapi, the national animal of the Congo. As for flora, an important species is Mangongo, whose leaves are used by the Mbuti, a pygmy ethnic group, to build their homes. The population is composed primarily of Alur, Hema, Lendu, Ngiti, Bira, and Ndo-Okebo, with differing figures on which one of the groups constitutes the largest percentage of the population in the province. The Mbuti reside primarily in the Ituri forest near the Okapi Wildlife Reserve, although some Mbuti have been forced into urban areas by deforestation, over-hunting, and violence. The Kilo-Moto gold mines are partly located in Ituri. In the beginning of the 21st century, petroleum reserves were found by Heritage Oil and Tullow Oil on the shores of Lake Albert.

**North Kivu** (French: *Nord-Kivu*) is a province bordering Lake Kivu in the eastern DRC. Its capital is Goma. North Kivu borders the province of Ituri to the north, Tshopo to the northwest, Maniema to the southwest, and South Kivu to the south. To the east, it borders the countries of Uganda and Rwanda. The province consists of three cities—Goma, Butembo, and Beni—and six territories—Beni, Lubero, Masisi, Rutshuru, Nyiragongo, and Walikale. The province is home to Virunga National Park, a World Heritage Site containing the endangered mountain gorillas. Except for the heightened insecurity and isolation due to rebel activities, North Kivu shares similar demographics with Ituri. The province is politically unstable and has been one of the flashpoints of the military conflicts in the region since 1998.

The **2018 or 10**^**th**^ **Kivu Ebola outbreak** began on August 1, 2018, when it was confirmed that four cases had tested positive for Ebola virus in the eastern region of Kivu in the DRC^9,10,11^. The Kivu outbreak included the Ituri Province, after the first case was confirmed on August 13^12^. This outbreak started just days after the end of the 2018 Équateur province Ebola virus outbreak of the DRC^13,14^.

The affected province and general area are currently undergoing a military conflict, which is hindering treatment and prevention efforts. The World Health Organization’s Deputy Director-General for Emergency Preparedness and Response has described the combination of military conflict and civilian distress as a potential “perfect storm” that could lead to a rapid worsening of the outbreak^15^. Due to the deteriorating situation in North Kivu and surrounding areas, the World Health Organization, on September 27, raised the risk assessment at the national and regional level from “high” to “very high”^15^.

The study population comprised adults aged ≥18 years living in the community, as well as the response team members. A 2010 estimate put the population of North Kivu at 5,767,945. An estimate of the population at 70% yielded 4,614,356 individuals. With an annual growth rate of 3.2%, the population in 2019 was put at 7,658,406 and 5,360,884 for the general public and those aged ≥18 years, respectively, in North Kivu. In contrast, a 2005 estimate put the population of Ituri at 4,037,561. An estimate of the population aged ≥18 years at 70% yielded 2,968,865 individuals. For 2019, the population was estimated at 6,275,305 and 4,392,714 individuals for the general public and those aged ≥18 years, respectively.

The response team consisted of over 10,000 persons. These individuals were in different response pillars, namely surveillance, risk communication, social anthropology, and vaccination. Other pillars included infection prevention and control, treatment and care, safe and dignified burial, as well as security, logistics, and administration, among others.

### Sample Size Estimation and Sampling Strategy

#### Sample size

This was an exploratory study. However, to achieve statistical conclusions on certain indicators of perceptions and practices juxtaposed on relevant demographic characteristics, a sample of the study population was taken. With an assumed 50% chance of having accepted Ebola control interventions at a 95% confidence interval with an error margin of 5%, a sample size 384 was computed for the quantitative study. For the two provinces, this yielded 768 participants. This was rounded up to 800 to make allowance for losses. The sample size of the qualitative study depended on the saturation of information after an initial pair was collected from each category of respondents.

#### Sampling strategy

A multi-stage sampling technique was adopted in selecting the communities, households, and respondents in this study. Two administrative areas (epicenters of the EVD outbreak within each province) were purposively selected. Ten communities were randomly selected from each of the two administrative areas in the province.

#### Selection of the household and respondents

The center of the selected community was the reference point from where the team spun a pencil to determine the first route and household; thereafter, they moved to the right to pick the next household and then continued until the number of households to be sampled was reached. Where there was a *cul-de-sac*, the step was retraced, and a turn to the left and then to the right was made to continue the sampling process.

Once in a selected household, an adult (≥18 years) was randomly selected for inclusion as a participant in the study. The sex of the participants was carefully alternated. For instance, if in household number one, a male was selected, a female was selected in the next household.

### Methods

This study was conducted using a mixed methods approach of qualitative and quantitative techniques. The methodology for data gathering included in-depth interviews (IDIs), focus group discussions (FGDs), and a survey using structured questionnaires. This type of study requires a strong focus on individual actors rather than state actors^16^.

### Techniques of Data Collection

#### FGDs

These were conducted in North Kivu and Ituri provinces.

Community leaders include political, traditional, religious, and social opinion leaders.

A set of questions covering different thematic areas was developed to guide the discussions. The questions covered healthcare services in the community, awareness of, and practices regarding EVD, as well as an assessment of the different pillars of the response interventions.

For the FGDs, eight to twelve persons were selected for each session. A minimum of two FGDs were conducted in the selected communities. There were separate FGD sessions for males and females in each of the communities. Overall, eight FGD sessions were conducted in each province.

**IDIs** were conducted in each community where the FGD was carried out. The IDI was held with community/opinion leaders in the selected communities and the team leaders of response pillars. Interviews were used to explore people’s opinions, views, and attitudes regarding practices and insights into the outbreak and response, as well as other socio-cultural factors that may influence their attitudes toward the response. The FGD guide was used for the IDI, focusing on the thematic areas of interest for the evaluation.

**A structured questionnaire** was used for collecting quantitative data from households. The questionnaire addressed all the indicators that were used for answering the research questions. The questionnaire was structured with results from the qualitative study. It was categorized into sections including socio-demographic data, perception of health problems in the community, knowledge of EVD, perceived epidemiology of Ebola in the communities, and sources of information on Ebola. Other areas included issues on communication and community engagement, infection prevention and control in the communities, vaccination, surveillance, and treatment and care. Other sets of questions covered safe and dignified burial, psychosocial issues, and logistic and security issues.

All interviews and discussions were tape-recorded, and detailed notes taken simultaneously, including verbal citations. Tape-recorded interviews were transcribed according to standard rules. Observations were also recorded and the discussion and interviews were triangulated with the quantitative data to arrive at conclusions.

#### Training and Pilot Trials

All instruments were translated into Swahili and French, the common languages spoken in the communities, and back translated to English for clarity of meaning. In each province, ten research assistants with substantial experience in community interactive research and the use of qualitative and quantitative techniques, as well as cultural sensibility, were recruited and trained for three days in Beni, and another three days in Bunia on the study objectives and use of the instrument for data collection. Training also included data entry into the ATLAS. ti template (qualitative data) and Epi Info (quantitative data). The instruments were reviewed after training for clarity, understanding, and sensitivity. Each province had a supervisor who worked with the principal investigator on data quality monitoring, safety advisory, and ethical conduct of the research, including the management of informed consent procedures. The study was conducted first in Ituri, then in North Kivu. The lessons learned from Ituri were used to manage the process in North Kivu, a more security- and logistics-challenged province. A data analyst developed and pre-tested the template for data entry and analysis using the pilot test output. Given the short period of the study, data were collected using pencil and paper instead of an android device. Fieldwork took 20 days to complete in each province before the analysis and report writing.

### Data Management

All **quantitative data** were double-checked by the researcher before entering in the computer. Data were entered into Epi Info and processed using SPSS. Descriptive statistics were used to determine the proportions of various categories of respondents, indicators, and to make comparisons. Frequency tables and graphic illustrations were used for presenting the data.

**Qualitative data** obtained from FGDs and IDIs were transcribed from audio recordings to text. All textual data were analyzed using the ATLAS.ti software package. Data were analyzed according to themes corresponding to the indicators in the quantitative data and triangulated during presentation to enable complementary and analogous interpretation.

Given the continuous analytical process involved in qualitative analysis, it is important to note that the initial analysis of the key informant interviews and FGDs informed the final development of the structured questionnaire to be used in the study. This further enhanced triangulation between the two sets of data to be collected. While the quantitative results gave us statistical conclusions, the qualitative results placed emphasis on what was actually said, providing illustrative quotes that gave context and depth to the quantitative results.

### Ethical Considerations

The principle of do-no-harm was adhered to in the study. Informed study approval was obtained at the levels of the province, local administration, community, and the households, while informed consent was obtained from all individuals involved in the study. The WHO/AFRO Ethics Review Committee provided ethical approval for the study. All researchers attended the mandatory training, which included substantial discussion of the ethical issues in research. Nearly 50% of the research assistants were women, ensuring same sex interviews and moderation of FGD sessions. The assistants were also trained and mandated to comply with child protection and gender sensitivity in the process of data collection and visits.

## Results

### Discharge from the ETC

Discharge was always an eventful moment. Some interviews were conducted in the car while the recovered waited for the final discharge arrangements to be made before leaving the ETC. During these interviews, family members were also present, which did not make things easier. Some of the former ETC patients had been to several other facilities before being admitted there. Following the first symptoms of infection, a man from an artisanal mining zone told us that he first went to a hospital, then to a health center, and finally to a health unit. He did all of this before going to his family in Mambasa. Compared to the other former ETC patients that we met, this was an unusual case. Most of those who recovered treated themselves at home before self-medicating with medicine from the pharmacy. They had typically considered their illness to be poisoning and resorted to a traditional healer before being taken care of by the Ebola response teams.

As they were leaving the ETC, the recovered were satisfied on many levels and said that their greatest satisfaction involved being cured. All of them were pleased with the treatment they were given, including the food, although some thought that the food was often not adapted to their eating habits. Overall, however, people were thankful for the medical and psychosocial care they received at the ETCs.

### Return to the Community

After people returned to their communities, the interviews were richer. Some of the former patients had many people who depended on them. One of them lived with his 15 children (eight boys and seven girls), two wives, and mother-in-law, and his return at home was highly cherished. However, a family member reported that this participant did not appreciate the celebrations at the ETC, where music was played as he left, because it drew too much attention and was the reason why others found out that he had been at the ETC. According to him, others believed that he was traveling. A healed woman from Butembo said that her family weas not willing to come see her leave the ETC where she was dancing with joy.

The opinions on the elements discussed converged among the recovered people leaving the ETCs. They were pleased with their recovery and claimed to have overcome EVD. A recovered person from a district in Mambasa said: *“I was satisfied with the ETC because I was cured. I was given advice before leaving. Besides, I was given clothes, including a blanket, a bed sheet, 60 kilograms of rice, five liters of oil, four bars of soap, one bag of salt, and one box of condoms.”* He also mentioned his satisfaction concerning the food because, according to him, the patients were given everything they asked for. They had breakfast at seven o’clock in the morning and lunch at noon.

A former patient from Butembo said: *“I was satisfied with the care, otherwise I would not have healed. They gave me credits to make phone calls. I was also given food and clothes.”* People who had recovered also appreciated the welcome they received as they arrived at the ETCs. One of these people, from Butembo, said that she had too much to eat—four meals a day. Another said that the ETC gave her hope to heal.

Those who recovered also received a lot more information about EVD during their stay at the ETCs. One former patient, who was a healthcare worker, said: *“It was after falling ill that I got a lot of information about Ebola at the ETC. I learned a lot of things about prevention: hand washing, wearing gloves, changing gloves before each patient, the need to wear work gear, how to measure temperature, keeping distance from the patient, sorting patients, isolating patients, and all the prevention measures. I caught the disease because I took care of a patient in a healthcare center without the necessary precautions.”*

The recovered talked about what their experience at the ETC meant to them. Every person we met had heard rumors about EVD, the ETC, and the vaccine. This had an impact on some of them during their stay at the ETCs and almost became an obstacle to the acceptance of the center as a place for treatment. A recovered man from a South African neighborhood in Mambasa said: *“I had been told that as soon as you enter the ETC, they would cut off your genitals or other body parts. No wonder people are afraid to go to the hospital. Some people say you get pricked and injected with a syringe and then you die. That is why people are afraid of the ETC. I learned all this before falling ill. So, I was scared. After I went to the ETC, I realized that everything I had been told was fake.”*

When the recovered returned home from the ETC, others visited them to learn about the ETC. That is why, upon returning to his community, a man was asked a series of questions by others from his community. He said: *“Many people from the neighborhood came. They did not stay long. Generally, they came to ask me questions. They asked if they had cut off body parts at the ETC. I told them it was a lie. I told them that my return to them was the proof. Some asked me if having headaches meant one has Ebola. I said that no one could know if the person had not been tested, and that the testing happens at the health center. I told them that once you get the test results, you are free to go home if you are negative for Ebola. I could not answer all the questions as they did not tell me much about certain subjects. It was the men who were asking me all these questions. Some women were there, listening, but not asking questions. Once a group of visitors left, another would come. The visitors would often stay for 30 minutes asking me questions. When the questions were about Ebola or the ETC, I had the answers. I said that I had agreed to go to the ETC and I learned a lot of things; I learned that the rumors about the ETC being a place where they cut off body parts of the sick were completely fake. After everyone left, the close neighbors came. They asked fewer questions. They said they would let me rest and come back the next morning. They did as they said.”*

Some former patients, such as healthcare workers, had caught the disease through their professional activities. Others were infected because of a relative, neighbor, or friend who was sick with EVD. One survivor said: *“I was taking care of my sick cousin. I would touch her and wash her clothes; I would throw away her vomit and other fluids. That is how I caught Ebola.”*

Those who recovered were much more informed about EVD than the average person. A recovered woman from Butembo said: *“Ebola is a terrible disease. It is very dangerous. It has killed our relatives. It almost killed me. Regarding transmission, I must say, it is a disease of contact. If you have contact with someone who is already sick, and you do not respect the prevention measures and the hygiene measures, you will become contaminated; if you do not get vaccinated, you run the risk of catching the disease. Besides, it is a sexually transmitted disease. For people who have recovered, unprotected sex can lead to contamination. We learned that at the ETC. You can also become infected by fluids from someone who is already sick, such as saliva, stool, urine, vomit, sweat, tears, vaginal secretions, and semen.”*

An EVD survivor from Butembo told us how she shared knowledge about the disease with members of her community who did not believe that EVD existed. When we asked if she knew people who did not believe in the existence of the disease, she replied: *“I know many. When I left the hospital, others would say: ‘Do you think you have Ebola? It was not Ebola, it kills in 30 minutes, you want to believe in this Ebola thing, Ebola, what is Ebola like, how does it happen?’ I told them I had Ebola. Not even my friends believed that I had Ebola. They told me: ‘All the others died there, but you came out well.’ They wanted me to stay there, maybe to die. They believed I was well-known there and that is why these people had decided to send me back home. I told them that nobody kills people at the ETC. It is the people who have not been there who believe that they kill the ill.”*

### Perception of Community Members

EVD survivors interacted with their community members about the disease and ETCs. They were confronted with rumors, and many community members who did not believe in the existence of the disease challenged them with questions, which they had to answer. After returning to their families, some of their relatives even asked them questions such as whether they could touch the recovered.

A recovered man from Mambasa said that the community members did not distrust him; instead, they came to him for information. However, he knew that not all the neighbors came. He did not experience any signs of marginalization or stigmatization from the other community members. Further, some of the recovered did not cut ties with the ETCs after they returned home. One said: *“I left six people at the ETC: four women and two boys. I hope they will come out alive like me. I went to visit them the day after I returned home. I was able to see them.”*

Most of the recovered suffered from stigmatization in their families and communities. Some community members did not believe in recovery and stated that they would have preferred it if the former patients were kept somewhere else after their discharge from the ETCs. Many of the recovered said that the youth are the most suspicious and that some of them say offensive things. One recovered person told us that she started to be called “mama Ebola” in her neighborhood and her children were referred to as “mama Ebola’s children.”

Stigma is also enhanced by a lack of information. Even some village chiefs lacked information and justified stigmatization. The chief of a camp in the Mandima health zone said the following: *“I think it is difficult to accept living with a person who has suffered from Ebola and was healed, as this person is coming from a place of care. We must first observe their condition and, if we notice they are not dangerous, we can accept them. But shaking hands will be difficult. In the community, we can always accept people recovered from Ebola, but eating with them using the same plate is tough.”*

The village chief of Bamako spoke of stigmatization through jokes like *Ebola man* and *Ebola woman*. However, he said that community leaders never make such jokes and are kind to those who have recovered. For the chief of the Madududu village, it was a great joy to see a person who had recovered and was returning in good health from the ETC, but this joy did not exclude fear: “*There is fear of living with a person cured from Ebola since we cannot trust them immediately; we can never know if they are suspicious or not. You do not know if the disease can come back. So, we have to keep our distance and observe what will happen*.”

The former patients spoke of the problems they faced upon their return to their communities, which included:

- Loss of work
- Loss of customers
- Loss of friends, in some cases
- Loss of the trust that people had in them
- The behavior of response agents. Some of the recovered spoke about the lack of ethics among response agents. They said that the behavior of certain agents was the reason for the loss of contacts, who often escaped from the response teams before arriving at the ETC. Frustrated, the contacts preferred to go elsewhere. A former patient, who is a health worker herself, said: *“When they contact someone who has started to show symptoms, such as fever, they tell them directly: ‘See, you have fallen ill*,*’ and this immediately frustrates the person, pushing them to go elsewhere, and change their living environment, to go where they can feel comfortable and free. This is why I talk about the need to change the behavior of people who are in the* riposte. *We recently had a case of a contact who died because he fled and got sick afterwards. All this happened because of their behavior with the contacts.”*
- Health problems after recovery
- Shame
- Frustration
- Marginalization and stigmatization

For many, however, these difficulties, especially the stigma, ended after a while. A recovered woman from the Boikene neighborhood in Beni said: *“The neighbors have totally changed now because we eat together; they are now convinced I am cured. They come to visit me, and we talk about various other topics besides Ebola.”*

Apart from providing information to community members informally during visits, patients were also involved in response activities. A traditional therapist in Beni spoke of three people who had recovered and found work at ETCs. They had sent them to the ETC. Others worked in response commissions, such as the “risk communication and community engagement commission.” This was the case for two of the people we met in Butembo. In Beni, some of the recovered had created an association to support the response activities.

One recovered woman was able to have her children vaccinated while she was still at the ETC. Other relatives were hostile to vaccination, but she was able to rely on her brother who was a doctor.

A recovered person from Butembo worked at the ETC as a nurse before working in a response team. Village and neighborhood leaders also talked about the involvement of the recovered in the Ebola *riposte* activities. The village head in the Mandima health zone said: *“Those who recovered from Ebola are becoming more and more influential. They also benefit from material support and their relationship with other community members is smooth. At the beginning, the community members were a bit resistant or afraid. With time, they began to understand and approach the healed without any problem.”* Problems encountered by the recovered included stigma and lack of social and professional integration.

### Stigmatization of the Sick and the Recovered, and the Desire to Hide the Sick

Healing is the purpose of caring. Recovery from an illness implies the return to one’s community, but this return is not free from trouble. Almost all the people who had been cured experienced stigmatization and marginalization to some extent. Their discharge became an event, with the mobilization of the community leaders. However, the reception back in the family was not always whole-hearted. A recovered woman said: *“Part of my family welcomed me with joy, but the other part avoided me and refused to approach me. They were afraid of me and so were the neighbors. I live next to a school. The children would come and look at me and say, ‘There she is.’ There were always many of them. I was an object of curiosity for everyone. This was very frustrating. When my younger sister went to the spring, everyone ran away from her, everyone ran away. People were saying that the Ebola families had come. My in-laws had taken my children while I was at the ETC. The children came a week later. They were happy to have me back.”*

Another woman went through a similar experience. She said that her neighbors were afraid and refused to visit her. According to her, at the market and at the spring, people were afraid of her. She added: “*They said ‘This is the Ebola mother.’ My children had taken the name of Ebola*.” She said this situation lasted for at least three months and the church was the only place where she was not stigmatized. Very few of those who returned from ETCs had not suffered from stigma. A high-ranking health professional was the only one to say he did not experience stigma, but he provided reasons for this: “*I have not experienced stigma, but some people do not believe I was sick with Ebola. Some people say I was given money to declare I was cured from Ebola. Some people say I am cheating and that I was not sick with Ebola, but other than that, there actually is a kind of stigma. People do not want to buy things around our house, which is next to the market. Traders near our house have seen their customers abandon them. Some people have asked to cut us out.”*

According to the high-ranking health official mentioned in the paragraph above, the situation changed when his wife got sick. After she was cured, the stigma began, and it even affected their neighborhood. He got a job as a nurse at the ETC in Beni after he recovered. He was not stigmatized there because many community members knew nothing about him. The chief of Kyamase village spoke about the contribution of the recovered in raising awareness. He said that he advised people to go to the healthcare center if they were ill. Village chiefs and community leaders spoke about the people who had been healed and their homecoming. Many talked of the problems of reintegration in the communities because of stigma and the resolution of problems over time. The avenue chief of Kilo-Moto in Bunia told us about the community members’ initial mistrust of the recovered and, after some time, their acceptance.

Community members spoke of the support given to the recovered, which included the following.

- Psychosocial assistance with advice from the concerned response teams
- Medical follow-up for other diseases
- The provision of food and other services

According to the chief of the Ngadi district: *“For the people cured from Ebola, what I realize is that they come back from the ETC with some goods that they were given following their treatment, such as rice and beans. These are the goods we see when they come back from the ETC, and there are some who are supported by counseling. They tell the community members to go to the ETC if they are sick; it is the place to hope for a cure. Personally, I am telling everyone to go to the ETC because they treat people well. People are being cured.”*

**Figure 1:**
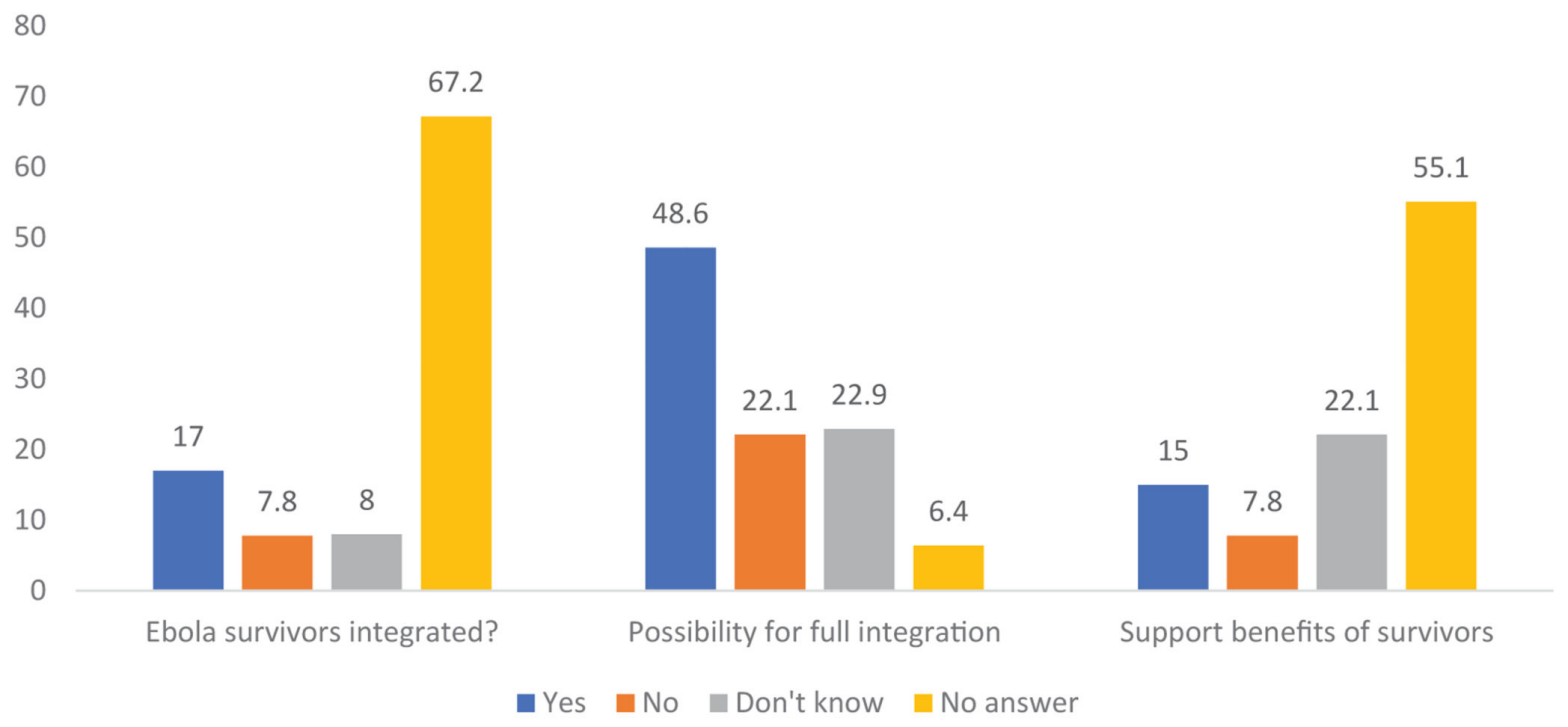
Awareness of integration of survivors and possibility of integrating survivors

The participants in the survey were asked if they were aware of the integration of Ebola survivors in the community. They were also asked if they think there is a possibility for full integration of Ebola survivors into the mainstream life of the community, as was the case before being infected. The responses to these questions are captured in Figure 1.

**Figure 2:**
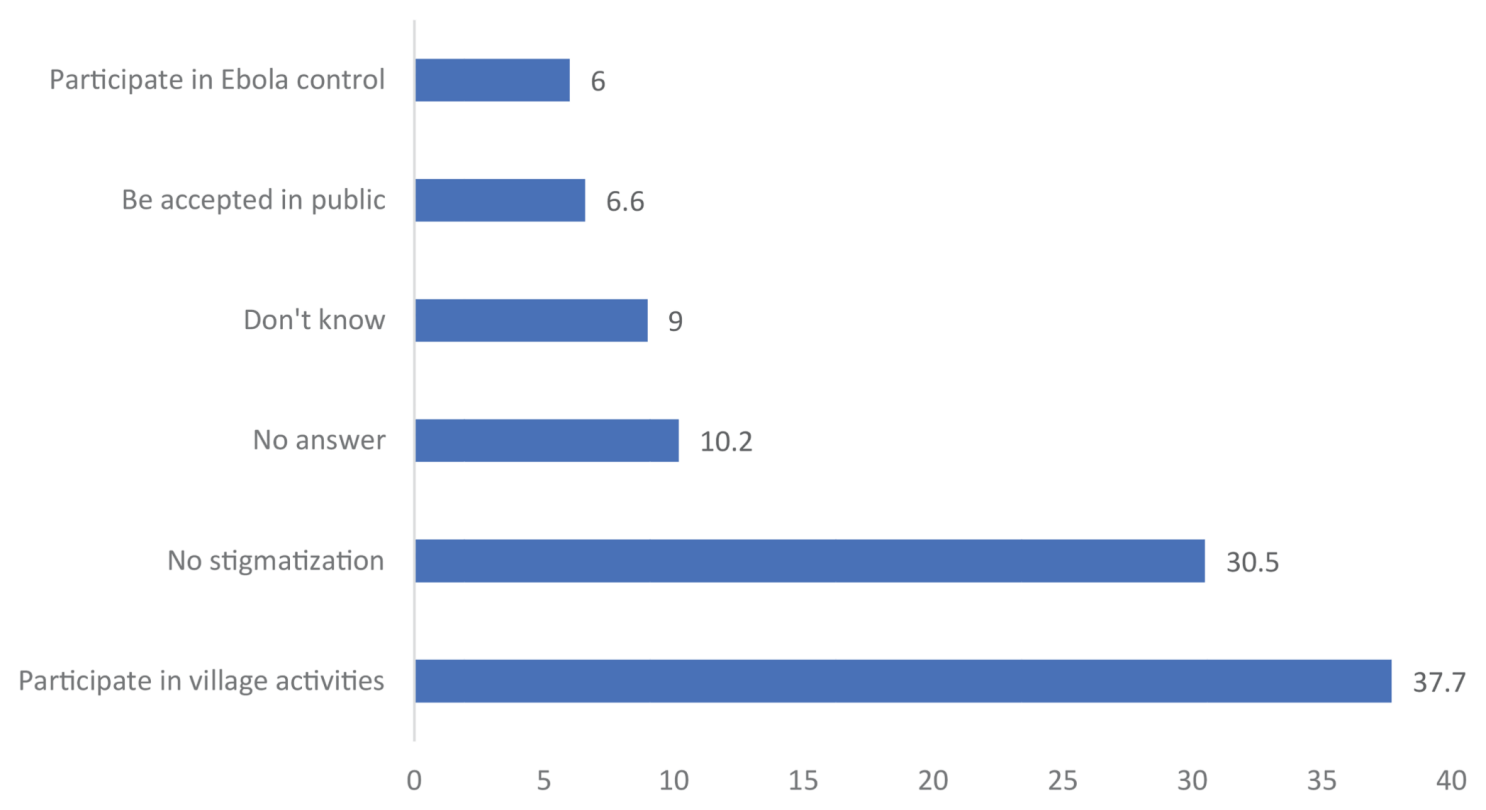
Ways of achieving integration of survivors

More than two thirds (67.2%) of the respondents could not confirm if Ebola survivors were integrated in the community. About 17.0% indicated that Ebola survivors have been integrated in the community. In contrast, a little less than half of the respondents (48.6%) indicated that it is possible to achieve full integration of Ebola survivors in the community life. Regarding the modalities for possible integration of survivors in the community life, Figure 2 revealed that 37.7% of the respondents think integration will be achieved by the participation of survivors in community activities. Slightly less than a third (30.5%) suggested that there should be no stigmatization toward Ebola survivors and 6.0% thought Ebola survivors could be engaged in EVD control programs. About 15% of the respondents thought survivors should be supported.

**Figure 3:**
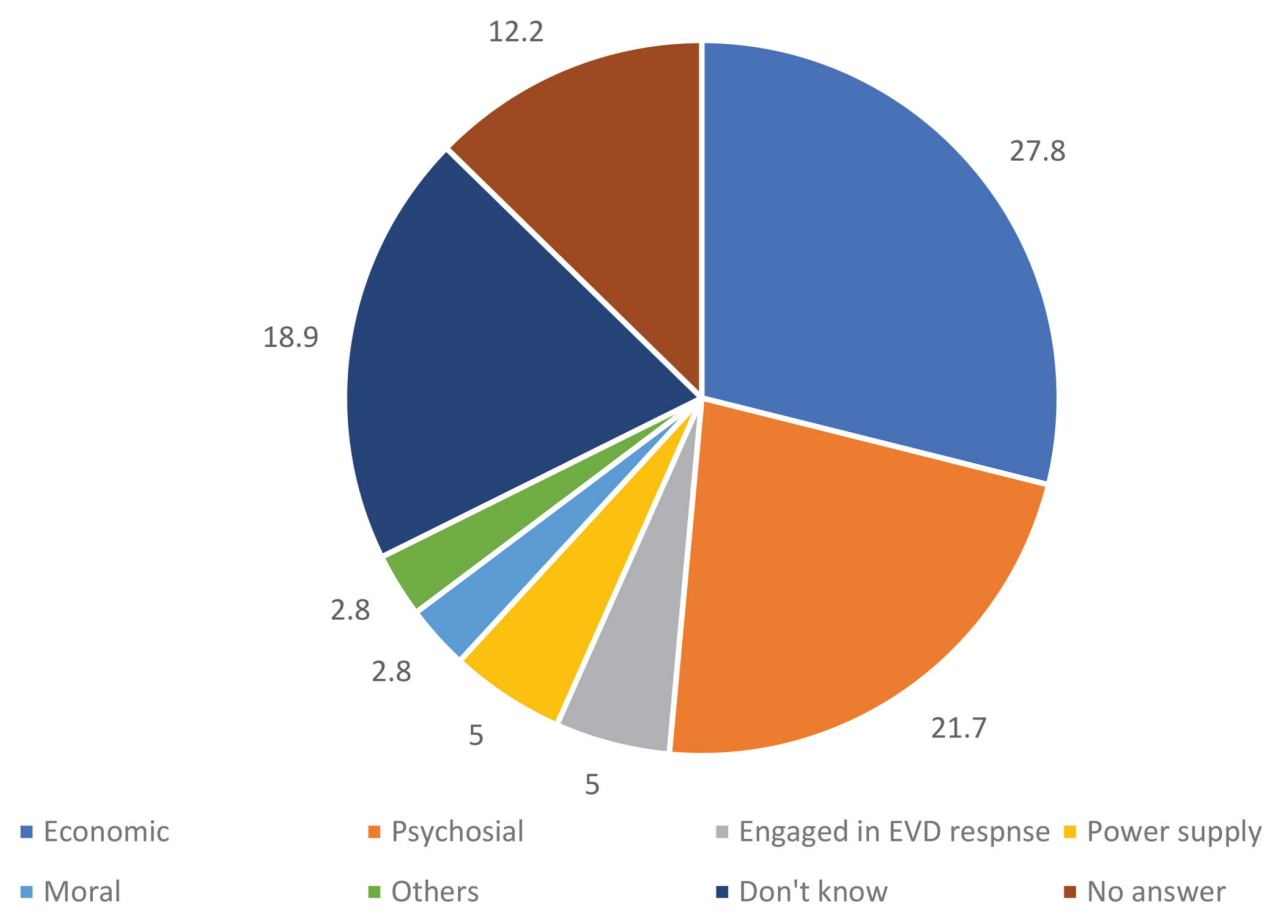
Possible support that can be given to a survivor in the community

Regarding the types of support that should be given to survivors, if any, the respondents mentioned economic support (27.8%), psychosocial assistance (21.7%), and power supply (5.0%), among others. The details can be seen in Figure 3.

**Figure 4:**
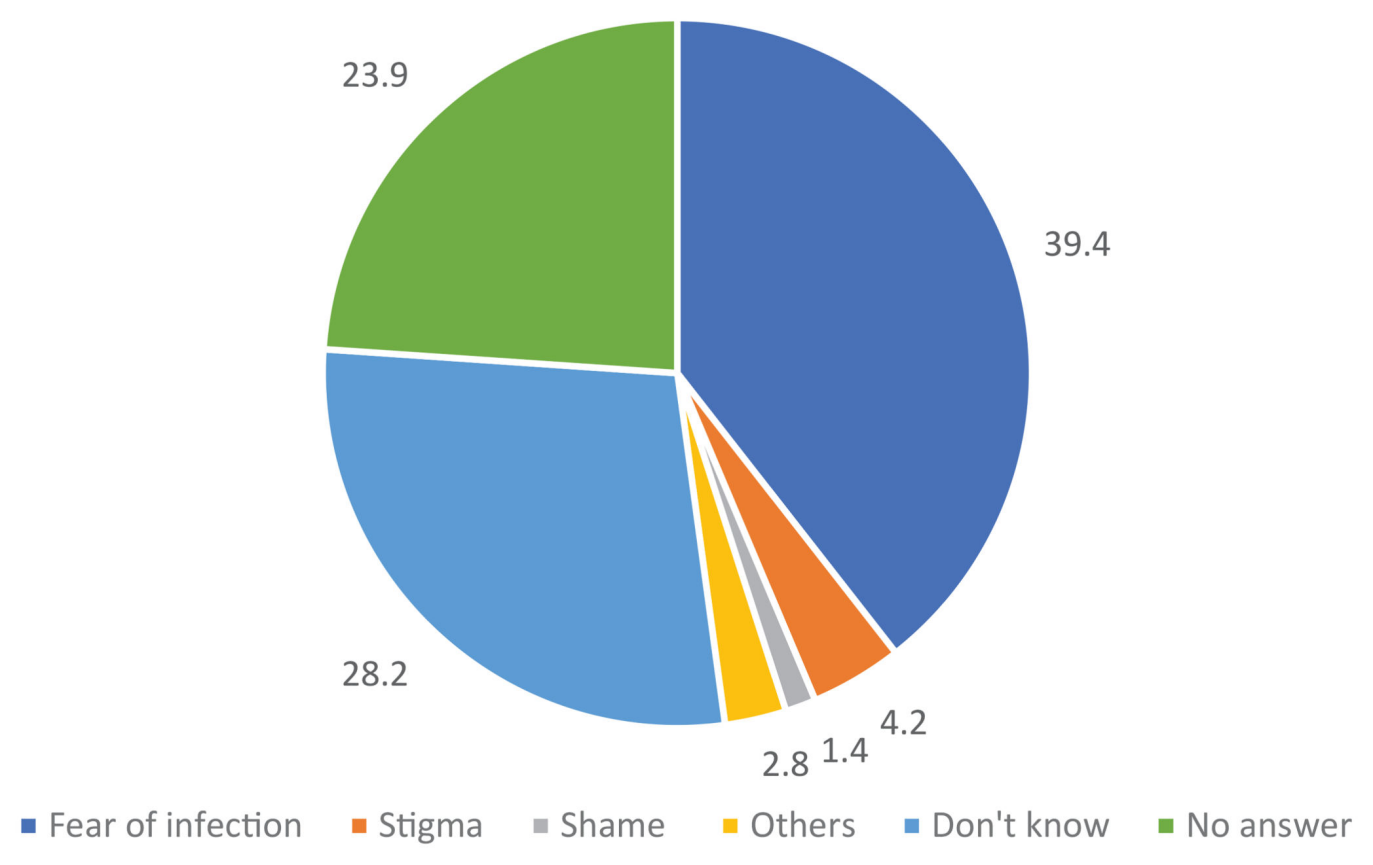
Possible reasons for the non-integration of EVD survivors

When asked for the possible reasons why Ebola survivors may not be integrated in the community life, more than a third (39.4%) of the respondents mentioned the fear of being infected by survivors. Other smaller proportions indicated stigmatization of the survivors (4.2%) and shame among them (1.4%). See Figure 4 for details.

Those who believed that the recovered were not well integrated thought that the community members were afraid of them, while more than a quarter gave no justification. These figures reflect a level of information.

### Proposals for the Recovered to Improve What Is Being Done in Terms of Caring for the Sick

The recovered made the following proposals for improving what is currently being done in terms of patient care:

- Vaccination of community members
- Awareness and provision of more information for the recovered when they leave the ETC
- The involvement of the recovered in the sensitization of community members
- The testimony of the recovered to inform other community members
- Communication in local languages to deal with resistance
- Information in churches and mosques
- Greater economic support for those recovered from EVD
- The separation of the sick from the other members of the community
- The limitation of certain information given to patients: People believe that certain information contributes to the stigmatization of those who have recovered, particularly regarding the presence of the Ebola virus in semen
- Care for the children of Ebola widows
- Care for Ebola orphans

## Discussion and Conclusion

Response to a disease occurs through treatment and care for patients as soon as they are given a positive diagnosis, with recovery being the desired result. Although the Ebola epidemic in the DRC has caused numerous deaths, many patients have been cured. For them, the experience of treatment has been beneficial and they have learned a lot about the disease. We met with people who had recovered once they were discharged from ETCs. At such meetings, the recovered were accompanied by their families and usually discussed the details of their illness and treatment on their return to the community. After leaving an ETC, the recovered were often busy and surrounded by family members, which made our interactions with them difficult. The day of discharge was transformed into an event by the media, which did not please some former patients. According to them, it contributed to publicizing the fact that they were ill.

Regardless, these former patients were generally happy that they were leaving the treatment center, essentially because of their recovery. When they returned to their families, the recovered had more time to talk about their experience. The ETCs had usually not been the first place they had visited in their quest for treatment, with some of them visiting places such as hospitals, health centers, and health posts. Before being admitted to an ETC, they were with their families and were trying different forms of treatment such as self-medication, traditional medicine, and modern medicine.

As for the ETCs, people were satisfied not only with the treatment itself, but also with the food and the way they were taken care of. The recovered, including those who were healthcare workers, learned more about EVD at the ETCs. The satisfaction they felt came from the fact that their entire experience had dispelled all the rumors they had heard regarding the ETCs. These rumors had been the source of the anguish they suffered when they were first admitted to the ETC, as they had been told that they would be subjected to degrading treatment and that their body parts, especially their genitalia, would be removed, which did not happen. They took advantage of the questions their visitors asked them to refute the rumors about ETCs, as these rumors had fueled their own fear of admission.

Overall, the recovered were satisfied with their treatment experience, except for a few who were not particularly pleased with the food. Through their experience, the recovered became a valid source of information for their neighbors and community members. The latter would come to them to verify information they had been given and to fact-check specific rumors. Thus, the recovered indirectly contributed to the response activities, especially by providing information to members of their communities. While they proved that healing was possible, their reintegration was not free of difficulties. They faced stigmatization by other community members and some even reported that there were health workers who were afraid of them following their recovery.

They also suffered the consequences of stigma, such as a loss of clients and work, of friends who stopped seeing them, and of self-confidence, shame, and frustration. Generally, those who recovered were provided with support, which included psychosocial assistance, medication, basic needs, and other material goods. The recovered contributed to the response by working as nurses in ETCs and by participating in the information dissemination activities of the response teams. They organized themselves into associations to better contribute and were efficacious. In some cases, they even organized sub-committees for better involvement in the response activities.

The recovered are proof of the effectiveness of the treatment provided in the ETCs. They gained knowledge from their experiences while being ill and the treatment they received for the disease. In most cases, discharge from the ETC was eventful and highly publicized. Very few of the recovered were available for an interview as they were leaving the ETCs, but they were all very satisfied with the treatment and their overall experience at the ETCs. The biggest reason for their satisfaction was that they were cured of the disease. For many of the former patients we met, the ETC was not the first place they went to during their quest for treatment. Some resorted to different types of health facilities such as hospitals, health centers, and health units, while being close to their families before being admitted to an ETC. From the interviews, we established that they had tried self-medication, traditional medicine, and modern medicine.

Recovered people (both men and women) have been subject to stigma in Sierra Leone and have often been rejected by their own families, in addition to losing their former activities and jobs. Stigma has also affected their families^17,18^. In Uganda, the recovered experienced stigma when they returned to their communities as they felt rejected in the supermarket, at the well, at the fountain, and while walking around the neighborhood. Women felt more stigmatized than men^19^. The recovered benefited from their experience at the ETCs and learned more about EVD and became a source of information for community members, as mentioned above. However, in previous epidemics, such as the one in 1995, some of the people who had recovered still considered the disease as a punishment from God, while others accepted that it was preventable^18^.

After leaving the ETCs and returning home, the former patients talked about their experiences during the treatment of the disease. They were all pleased to be cured and satisfied with the way they were taken care of at the ETCs. In general, they were very satisfied with the information they received about the disease at the ETCs, which was even the case for healthcare workers who were admitted to an ETC after falling ill. Rumors about the disease and ETCs had been a source of anxiety as admission to the ETC was presented as a probable path toward death. Recovered patients spoke of the constant fear they experienced as they thought they would undergo the removal of their organs or body parts. They were satisfied with all the attention and treatment they received. Only a few mentioned the food, which did not always correspond with what they usually ate. Overall, they were satisfied.

After returning to their communities, and just like the rest of the community members, former patients used healthcare services, self-medication with modern products, and self-medication with traditional medicines to deal with their health problems^5,20,21^. Those who recovered benefited from the care of modern healthcare workers. For these professionals, the recovered individuals have the same reasons for a consultation as any other patient. However, these health professionals faced problems such as a lack of medicines, the impossibility of running certain tests, and poor coordination of referrals from peripheral health facilities^8^. The recovered still elicited a certain amount of fear among some modern medicine providers. Cases of refusal of treatment were highlighted and health professionals were pleased if they could simply provide a prescription^8^. Apart from these few cases, healthcare professionals were available to provide care to those who survived EVD. However, many opted for traditional medicine because of the accessibility and affordability of traditional medicines or due to the influence of relatives or peers.

Upon returning to their families, the recovered became the source of information for their neighbors and other community members. Their visitors not only came to welcome them back to their homes but also to ask them questions about their stay at the ETC and their treatment, to verify information, and to fact-check the rumors they had heard about the disease, its treatment, and the ETCs. The former patients helped to provide information on the treatment of EVD, especially on the reality of infection and the possibility of treatment and recovery, for which they were living proof.

The reintegration of the recovered in their environment of origin was not difficult in most cases. However, some faced stigmatization by members of their community, which resulted in loss of clients, loss of work, loss of friends who stopped seeing them, and loss of confidence in themselves. Some said that even healthcare workers avoided them. This situation was a source of shame and frustration, and fear of stigmatization was the leading cause for family members wanting to hide patients and make them abandon treatment.

However, in general, the former patients benefited from the various forms of support that they received, including psychosocial assistance. They were also given medicine, food, and other material goods. The recovered played a significant role and contributed to the response to the EVD outbreak. Some of them even created an association in various sub-coordinating offices to contribute to the response amidst the EVD epidemic. Such support services have helped people reintegrate and adjust to their respective communities, as shown in other studies^22,23,24^. This has particularly been true for female survivors^23,25^.

Despite its findings, this study was not devoid of limitations. One such limitation was related to its timing. The study was conducted during the EVD outbreak with the hope of generating data to guide the design of the response. During the outbreak, there were cases of resistance to the response team because of many misconceptions. All of these affected the depth of assessment of the data, particularly qualitative data, which should provide more epistemological evidence of the circumstances around the outbreak and the consequent interventions. While this study regarding the outbreak provides data to shape suitable responses, a post-outbreak survey will provide a more rigorous interrogation of the issues.

## Figures and Tables

**Table 1:** Distribution of participants in the IDI and FGD sessions by provinces

Target	North Kivu				Ituri Province			
	Butembo		Beni		Mbuti		Bunia	
	IDI	FGD	IDI	FGD	IDI	FGD	IDI	FGD
Pillar leads	All		All		All		All	
Pillar members	2/pillar		2/pillar		2/pillar		2/pillar	
Community leaders^1^	≥2/community		≥2/community		≥2/community		≥2/community	
Leader of survivor group	≥2/community		≥2/community		≥2/community		≥2/community	
Community adult males		≥2 groups		≥2 groups		≥2 groups		≥2 groups
Community adult females		≥2 groups		≥2 groups		≥2 groups		≥2 groups
Community male youth		≥2 groups		≥2 groups		≥2 groups		≥2 groups
Community female youth		≥2 groups		≥2 groups		≥2 groups		≥2 groups
Survivors		≥2 groups		≥2 groups		≥2 groups		≥2 groups

## Data Availability

The data that support the findings of this study are not publicly available as they contain information that could compromise the privacy of the research participants. The data are available from the corresponding author (Joseph Okeibunor) upon reasonable request.
